# Surgical Therapy of Esophageal Adenocarcinoma—Current Standards and Future Perspectives

**DOI:** 10.3390/cancers13225834

**Published:** 2021-11-21

**Authors:** Wolfgang Schröder, Suzanne S. Gisbertz, Daan M. Voeten, Christian A. Gutschow, Hans F. Fuchs, Mark I. van Berge Henegouwen

**Affiliations:** 1 Department of General, Visceral, Cancer and Transplantation Surgery, Faculty of Medicine and University Hospital Cologne, University of Cologne, 50937 Cologne, Germany; Hans.fuchs@uk-koeln.de; 2 Cancer Center Amsterdam, Department of Surgery, Amsterdam UMC, University of Amsterdam, 1105 AZ Amsterdam, The Netherlands; s.s.gisbertz@amsterdamumc.nl (S.S.G.); d.voeten@amsterdamumc.nl (D.M.V.); m.i.vanbergehenegouwen@amsterdamumc.nl (M.I.v.B.H.); 3 Department of General and Transplantation Surgery, University Hospital Zurich, 8091 Zurich, Switzerland; christian.gutschow@usz.ch

**Keywords:** esophageal adenocarcinoma, transthoracic esophagectomy, minimally invasive (robotic) techniques: perioperative management, patient selection, surgical outcome

## Abstract

**Simple Summary:**

Subtotal resection of the esophagus with resection of local lymph nodes is the oncological procedure of choice for advanced esophageal cancer. Reconstruction of the intestinal tract is predominantly performed with a gastric tube. Even in specialized centers, this surgical procedure is associated with a high complication but low mortality rate. Therefore, clinical research aims to develop peri- and intra-operative strategies to improve the patient related outcome.

**Abstract:**

Transthoracic esophagectomy is currently the predominant curative treatment option for resectable esophageal adenocarcinoma. The majority of carcinomas present as locally advanced tumors requiring multimodal strategies with either neoadjuvant chemoradiotherapy or perioperative chemotherapy alone. Minimally invasive, including robotic, techniques are increasingly applied with a broad spectrum of technical variations existing for the oncological resection as well as gastric reconstruction. At the present, intrathoracic esophagogastrostomy is the preferred technique of reconstruction (Ivor Lewis esophagectomy). With standardized surgical procedures, a complete resection of the primary tumor can be achieved in almost 95% of patients. Even in expert centers, postoperative morbidity remains high, with an overall complication rate of 50–60%, whereas 30- and 90-day mortality are reported to be <2% and <6%, respectively. Due to the complexity of transthoracic esophagetomy and its associated morbidity, esophageal surgery is recommended to be performed in specialized centers with an appropriate caseload yet to be defined. In order to reduce postoperative morbidity, the selection of patients, preoperative rehabilitation and postoperative fast-track concepts are feasible strategies of perioperative management. Future directives aim to further centralize esophageal services, to individualize surgical treatment for high-risk patients and to implement intraoperative imaging modalities modifying the oncological extent of resection and facilitating surgical reconstruction.

## 1. Introduction

According to international and national guidelines, surgery is generally accepted as the mainstay of curative treatment for esophageal adenocarcinoma [1,2,3]. In the western world, the vast majority of carcinomas present as locally advanced tumors requiring some kind of a standardized multimodal treatment. Neoadjuvant chemoradiotherapy according to the CROSS protocol has proven to be beneficial and is currently the most common treatment strategy for locally advanced adenocarcinoma [4,5,6]. Perioperative chemotherapy according to the FLOT protocol has also demonstrated a survival benefit, although a significant percentage of patients do not complete the recommended postoperative cycles [7,8]. A direct comparison of these two protocols is currently under investigation in two European prospective randomized trials [9,10]. Despite advances in the medical and surgical field, long-term prognosis remains limited due to locoregional and distant recurrent disease with, reported 5-year overall survival rates not exceeding 47% for the CROSS and 45% for the FLOT protocol [5,8].

As the surgical part of the multimodal concept, transthoracic esophagectomy with gastric reconstruction is currently considered to be the gold standard for esophageal adenocarcinoma [11,12]. With the evolution of minimally invasive, including robotic, techniques, a huge bunch of technical variations exists for this complex procedure, and no consensus about the optimal approach has been established yet [13]. Despite the technical complexity and diversity, the demands on surgical quality and outcome in the context of multimodal strategies are increasingly high.

With respect to well-established prognostic factors of long-term outcome, the fundamental significance of oncological esophagectomy remains to achieve a complete resection of the primary tumor (R0 resection) and to perform an adequate lymphadenectomy (LAD), both in order to reduce the risk of locoregional or distant recurrent disease. In the majority of observational registry analyses, transthoracic esophagectomy yields a sufficiently high R0 resection rate usually exceeding 95% [14,15]. In contrast to squamous cell carcinoma, the extent of lymphadenectomy for esophageal adenocarcinoma is still a matter of ongoing debate [16]. As the nodal clearance of the abdominal and mediastinal compartment is performed by the majority of esophageal surgeons, it is not clear whether an extended lymphadenectomy of the upper mediastinum (extended 2-field LAD) or even the cervical compartment can improve long-term survival and outweigh the potential surgically related complications.

Irrespective of possible technical modifications, considerable postoperative morbidity and mortality have been reported for transthoracic esophagectomy, even in expert centers with a caseload above average [14,15,17]. It is evident that postoperative complications not only have an adverse effect on postoperative health-related quality of life but also display a strong impact on long-term oncological outcomes [18]. In a recent meta-analysis comprising 21 studies, postoperative pulmonary complications and anastomotic leakages were associated with a significantly decreased overall 5-year survival [19]. Therefore, an obvious and predominant aim of esophageal surgery remains to reduce postoperative morbidity and associated mortality. In order to achieve this goal, the scientific interest focuses on the surgical technique itself as well as the entire perioperative period, with three distinct fields of clinical research: the appropriate selection of patients for transthoracic esophagectomy, the preoperative conditioning of single organ dysfunctions (prehabilitation), and fast-track protocols during postoperative recovery.

This review aims to summarize the present evidence on surgical strategies currently applied for esophageal adenocarcinoma, but also to point towards unsolved questions associated with the surgical management of this tumor entity. Finally, a brief prospect will be given on possible future developments expected in this distinct field of surgical oncology.

## 2. Perioperative Management

### 2.1. Potential Risk Factors and Patient Selection

More than 80% of patients with esophageal adenocarcinoma receive some kind of neoadjuvant treatment prior to esophagectomy, and at the present it is not clear whether chemoradiotherapy and/or chemotherapy alone should be considered as a potential risk factor for postoperative morbidity. Several observational studies indicate that physical function deteriorates during multimodal treatment. In a prospective cohort study, the cardiopulmonary function was assessed using exercise testing in esophageal cancer patients before and after neoadjuvant chemotherapy and chemoradiotherapy [20]. For both regimens, the anaerobic threshold and peak oxygen uptake demonstrated a significant reduction, indicating a decline in the cardiopulmonary reserve following neoadjuvant therapy that was sustained up to the point of surgery at four weeks after chemoradiotherapy. A recently published systematic review summarizing data up to June 2016 confirmed the results of single observational studies that chemotherapy and chemoradiotherapy causes a general reduction in physical function [21]. On the other hand, in the latest meta-analysis of 2014 summarizing data from 23 clinical studies suggested that there was no evidence that either neoadjuvant chemotherapy nor chemoradiotherapy increase the risk of postoperative morbidity or perioperative mortality compared with surgery alone [22].These conflicting results between clinical interventional and postoperative outcome studies have not been sufficiently explained yet [23]. However, it must be considered that neoadjuvant chemotherapy itself is especially associated with a considerable preoperative morbidity, as demonstrated by grade five toxicity of 1.5% [23], and therefore a significant percentage of patients will not be scheduled for esophagectomy.

Sarcopenia, defined as a state of severe muscle loss and function, has been clearly identified as risk factor of perioperative morbidity, and primarily reflects the reduced nutritional intake often associated with esophageal adenocarcinoma. In a recent meta-analysis including 29 studies with more than 3000 patients, sarcopenic patients had a higher incidence of pulmonary complications [24]. However, different methods to assess the body composition (computed tomography and bioelectrical impedance analysis) are currently practiced, hampering interinstitutional comparisons. Two recent single-center studies investigated the longitudinal variations in body composition by computed tomography in patients undergoing esophagectomy [25,26]. Following surgery, patients lost on average 13% of their skeletal muscle, 65% of their visceral and 44% of their subcutaneous adipose tissue. In particular, male patients with a >10% decrease in the skeletal muscle index had a significantly lower 5-year overall survival. The authors concluded that the evaluation of the body composition has the potential to become a valuable clinical tool for preoperative risk assessment, with an impact on the clinical decision making. The data also underline the outstanding importance of perioperative nutritional support in this particular patient cohort.

In a recent meta-analysis summarizing data from 39 eligible studies, various patient-related factors associated with major postoperative complication and mortality were identified [27]. These included male sex, age > 70 years, American Society of Anesthesiologists (ASA) score > III, cardiac and renal comorbidities, diabetes and habitual alcohol usage. In addition to the identification of single risk factor for the postoperative outcome, several working groups have investigated a composite risk score in order to predict morbidity and mortality following esophagectomy and to select patients for the complex procedure [28,29,30]. Based on 24,000 esophagectomies collected in an American database (Nationwide Inpatient Sample, NIS), predictors of postoperative mortality were analyzed [28]. Minimally invasive techniques (including hybrid procedures), and operations performed in high-volume centers were protective, whereas increasing age, comorbidities and histology of squamous cell carcinoma were independent predictors of mortality. Using these simple variables, a set of sensitivity/specificity analyses defined low- and high-risk patients, which correlated with the observed postoperative mortality. The authors concluded that this risk scale possibly serves as a helpful tool for preoperative patient counseling.

The International ESODATA Study Group (IESG) database analyzed 8403 esophagectomies with an overall 30- and 90-day mortality rate of 2.0 and 4.2%, respectively [30]. Patients were randomly assigned to development and validation cohorts, aiming for a final scoring system that categorized patients into five homogenous risk groups predicting 90-day mortality. The multiple logistic regression model identified 10 weighted point variables factored into the prognostic scores for age, sex, body mass index, performance status, myocardial infarction, connective tissue disease, peripheral vascular disease, liver disease, neoadjuvant treatment, and hospital volume. On the basis of these preoperative variables, the IESG risk-prediction model allowed stratification of an individual patient’s risk of death within 90 days.

In summary, advanced age, the patient’s general condition, and the number of comorbidities remain the predominant predictors of a poor postoperative outcome and should be taken into account for the selection of patients undergoing oncological esophagectomy. The knowledge of validated risk factors and the appropriate selection process prior to esophagectomy is still the method of choice to achieve a low mortality rate. However, since a clear-cut score has not been established yet, the decision making in terms of esophagectomy is ultimately left to the surgeon’s personal experience and discussion with the patient at the preoperative evaluation.

### 2.2. Prehabilitation

Prehabilitation summarizes the concept of conditioning single-organ dysfunctions in the preoperative period in order to facilitate postoperative rehabilitation and to improve surgical outcome. For esophageal cancer patients, predominant organ dysfunctions comprise an impaired pulmonary function, a poor nutritional status and a reduced physical fitness with a compromised cardiovascular reserve. Due to the potential of prehabilitation concepts to reduce postoperative morbidity, this emerging field has gained increasing scientific interest. However, current literature demonstrates a significant heterogeneity between studies, with a broad variety of preoperative interventions, timelines and outcome measures reported [31]. So far, only a small number of prospective randomized controlled trials (RCT) were published between 2015–2021 [31,32]. The main interventions applied in these RCT are preoperative inspiratory muscle training [33,34], physical aerobic exercising [32] and nutritional guidance/supplementation [32]. Despite the heterogeneity of the published RCTs, prehabilitation concepts are feasible and have been proven to improve cardiorespiratory function as well as aspects of quality of life but evidence is still too weak to conclude on the reduction in postoperative morbidity following esophagectomy. Similar conclusions can be drawn from the low number of rehabilitation RCTs with interventions primarily focusing on postoperative physical training [35,36].

### 2.3. Fast-Track Protocols

Fast-track protocols (synonym: enhanced recovery after surgery, ERAS) intend to perioperatively maintain the physiological homoeostasis and thereby accelerate postoperative rehabilitation and reduce morbidity. Enhanced recovery after surgery (ERAS) protocols when compared to standard care pathways are gaining increasing interest in the perioperative management of esophageal cancer patients but have not reached the level of complete implementation, even amongst specialized centers yet.

In a recently published meta-analysis, four randomized controlled and four nonrandomized, prospective trials with a total of 1133 patients (599 ERAS and 534 standard pathways) were eligible for further analysis [37]. The overall morbidity, in particular pulmonary complications, the length of hospital stay as well as the total hospital costs were in favor of the ERAS pathway. However, existing protocols in different centers are characterized by a great variability, with a significant heterogeneity of the evaluated outcome parameters. To overcome this diversity of protocols, a consensus conference of international experts was assembled to review the current composition of ERAS pathways and the evidence of fast-track protocols [38]. In total, 39 sections covering the perioperative period as well as technical aspects of the esophagectomy itself were ultimately produced, demonstrating the broad spectrum of different components contributing to fast-track protocols. Despite advances in this developing field, the limiting variable of all ERAS protocols remains the early and adequate enteral feeding load of the gastric conduit, which is still a matter of an ongoing discussion.

## 3. Currently Practiced Surgical Techniques

For esophageal adenocarcinoma, the esophageal resection can be performed via a transthoracic or a transhiatal approach, and is usually followed by a gastric-tube reconstruction [39,40]. A transthoracic esophagectomy is performed through a thoracotomy, which allows for better visualization, a complete sharp en-bloc dissection of the esophagus and an extended thoracoabdominal lymphadenectomy [41]. During a transhiatal resection, the esophagus is resected via the hiatus esophagi and the thorax remains closed. Therefore, only an abdominal and lower mediastinal, paraesophageal lymphadenectomy is performed. Generally, the transhiatal approach is an alternative to the transthoracic procedure for primary tumors located in the distal esophagus or at the gastroesophageal junction. Transthoracic surgery is considered oncologically superior as a more extended, two-field lymph node dissection can be performed [42,43]. On the other hand, transhiatal surgery is associated with lower postoperative complication rates (especially a lower pulmonary complication rate) and a shorter hospital stay [42,44]. Next to surgeon preferences, the existence and location of lymph node metastases also plays part in the decision for either transhiatal or transthoracic surgery. To overcome the limited extent of lymphadenectomy, a combined cervical–transhiatal approach is being developed, but only a small series of preliminary results have been published so far [45,46].

There is only one randomized controlled trial that compared outcomes of transthoracic (right thoracotomy) and transhiatal surgery in patients with an adenocarcinoma of the mid or distal esophagus [47,48]. This trial failed to identify a survival benefit for either of the surgical procedures. However, the results of this study were published in 2002 in the pre-(neo)adjuvant-therapy and pre-minimally invasive surgery era, and requires current-day verification as multimodality treatment and minimally invasive surgery are widely applied nowadays. In recent years, transthoracic surgery has gained popularity, and currently over 80% of esophageal resections are performed via a transthoracic approach [14].

### 3.1. Hybrid vs. Total Minimally Invasive Techniques

Since the results of the TIME trial were published in 2012, minimally invasive esophagectomy has enjoyed increasing interest worldwide [49]. This trial compared outcomes of minimally invasive esophagectomy with its open equivalent in 115 patients treated in five European centers. While the quality of surgery was comparable between both operative procedures in terms of 3-year overall survival, surgical radicality and lymph node yield; pulmonary complication rates, blood loss, length of hospital stay and quality of life were significantly better after minimally invasive surgery [50,51].

The 2019 MIRO trial compared the open transthoracic procedure with a hybrid approach, in which the abdominal phase was performed laparoscopically and the thoracic phase open [52]. The study found significantly lower intra- and post-operative (major) complication rates after hybrid surgery. The literature suggests improved pulmonary complication rates after total minimally invasive surgery compared to hybrid procedures [53,54]. However, randomized, controlled trials comparing both techniques are lacking. Currently, the ROMIO trial is randomizing patients for hybrid and open esophagectomy; a substudy will also evaluate the safety of total minimally invasive surgery [55,56].

A third randomized controlled trial, the ROBOT trial, compared robot-assisted minimally invasive esophagectomy (RAMIE) with an open transthoracic procedure. The RAMIE approach consisted of a robot-assisted thoracoscopic phase and a laparoscopic abdominal phase [57]. Pulmonary and cardiac complication rates were significantly lower after RAMIE, and postoperative pain, functional recovery and quality of life were improved after robot-assisted surgery. Advantages in terms of postoperative (pulmonary) complications of RAMIE over conventional minimally invasive esophagectomy have been described in the literature [58,59]. The currently recruiting ROBOT-2 trial will provide randomized evidence on the comparison of RAMIE versus minimally invasive esophagectomy [60].

The superiority of either completely minimally invasive surgery, hybrid surgery or RAMIE over open surgery has been demonstrated in several observational clinical trials [51,61,62]. These trials have resulted in the widespread introduction of minimally invasive surgery in everyday clinical practice. In the Netherlands, for example, over 90% of esophageal resections are currently performed minimally invasively [63]. However, minimally invasive techniques are associated with considerable proficiency-gain curves [64]. Multiple population-based studies have failed to confirm the RCT-proven advantages of minimally invasive surgery over open procedures [65,66,67,68,69]. A recent study examined the implementation of minimally invasive surgery outside of the randomized controlled setting into national practice [70]. This study concluded that external validity of the TIME trial results into Dutch national practice was low, and that national introduction of the new technique resulted in an increase in pulmonary complications and reoperation rates. These results suggest that extrapolating trial results into a nonstandardized, noncontrolled and nonexpert national practice are associated with increased complication rates. This underlines the importance of extensive proctoring when implementing novel techniques, with considerable learning curves.

### 3.2. Anastomotic Techniques: McKeown vs. Ivor Lewis Esophagectomy

When performing a transthoracic esophagectomy with gastric tube reconstruction, the anastomosis can be placed in the cervical (McKeown procedure) or intrathoracic compartment (Ivor Lewis procedure). Next to disease specifications, such as the exact location of the primary tumor and possible location of lymph node metastasis, surgeon preferences play a large role in the choice for a McKeown or Ivor Lewis procedure. From the ESODATA database, in which 24 high-volume centers from 14 countries register their esophagectomies, it emerges that over 60% of anastomoses are placed intrathoracically [14]. The discussion on the optimal anastomotic location has been extensive. The general consensus that McKeown procedures lead to higher anastomotic leakage and resultant stenosis percentages, but that an anastomotic leakage after an Ivor Lewis procedure tends to have more severe consequences had much support for a long time [71,72]. Recently, the ICAN trial results were published [73]. In this RCT, cervical and intrathoracic anastomosis were compared following esophagectomy for cancer. After randomization of 262 patients, leakage rates were 12.3% for intrathoracic and 34.1% for cervical anastomosis. Length of intensive care unit stay, quality of life and mortality rates were comparable between the groups. However, less severe complications and lower recurrent nerve palsy rates were reported after Ivor Lewis surgery. Therefore, intrathoracic anastomosis should be the preferred anastomotic location after minimally invasive esophagectomy, but then again, the proficiency gain-curves and safe implementation of new anastomotic techniques should be thoroughly considered when implementing these trial results in everyday practice [64].

## 4. Postoperative Outcome

### 4.1. Classification of Postoperative Complications and Outcome

International literature on the surgical treatment of esophageal cancer is abundant and mainly focusses on decreasing the considerable postoperative mortality and morbidity. However, postoperative morbidity rates have been reported to be as low as 17% and as high as 74% [74,75]. Such heterogeneity in outcomes is unlikely to be solely caused by actual differences in clinical practice and outcomes. Differences in outcome definitions and documentation probably also play an important role. Therefore, standardized outcome measure definitions are of vital importance to validly compare outcomes between countries and institutions, review the effect of quality improvement programs, review the effect of novelties in treatment and to establish clear-cut international benchmarks for outcomes of esophageal cancer surgery. In that scope, the Esophageal Complications Consensus Group (ECCG) was established in 2011 and includes a large number of high-volume esophageal cancer surgeons from 14 countries. In several Delphi rounds and meetings, they developed the standardized ECCG complication definition set [76]. The standardized complication set includes pulmonary, cardiac, gastrointestinal, urologic, thromboembolic, neurologic/psychiatric, infectious and wound/diaphragm related complications. An example of the pulmonary and gastrointestinal complication set is presented in Table 1 [76]. Outcome definitions are presented in Table 2 [76]. Next to registering the occurrence of complications, the ECCG also promotes the registration of the severity of complications using the validated Clavien–Dindo grading system (Table 3) [77]. Several population-based, nationwide (audit) registries have already adopted the ECCG complication definitions in order to allow for uniform registration of complications and international comparison [78,79].

### 4.2. Morbidity and Mortality

The ECCG also maintains a database in which 24 high-volume centers from 14 countries register detailed esophagectomy information: the ESODATA database [14]. This database is considered as an important international benchmark. It reports surgical radicality rates (R0) of 93.4% and overall complication rates of 59%. The most frequently observed complications constitute pneumonia and atrial dysrhythmia, both at 15%. Anastomotic leakage is observed in 12% of patients, while chyle leakage and recurrent nerve palsy occur less frequently at 4.7% and 4.2%, respectively. The ECCG defines a severe complication as Clavien–Dindo grade IIIa or higher (Table 3), and the incidence of severe complications is reported at 17%. Postoperative mortality at 30 days occurs in 2.4% and in 4.5% of patients at 90 days postoperatively. As the occurrence of postoperative complications is associated with long-term overall survival, lowering complication rates is of the utmost importance [80]. Using ECCG definitions, the Dutch national registry reported higher postoperative morbidity with complication rates of 65% and severe complications occurring in 29% of patients [78]. However, postoperative mortality at 30 days was reported in only 1.7% of patients. The Irish national dataset even reported 30-day mortality rates of 0% and postoperative complication rates of 54% [79]. The large differences in outcomes between countries, even though similar definitions were used, provide very valuable information, as identification of differences in treatment strategies that cause these outcome differences may provide clear leads towards quality improvement [81].

Additionally, the identification of interhospital variation in clinical practice and outcomes of different hospitals within one country may pave the way towards nationwide quality improvement [82,83,84,85,86]. Especially in countries where hospital resources are comparable, it should not matter in what hospital a patient is being treated and every hospital should be able to perform up to the standard of the best-performing center. Disclosing and safe-sharing hospital results and discussing these outcomes with other surgeons has proven to be an effective tool towards quality improvement on a national level [87].

The use of composite outcome measures should also be promoted in esophageal cancer surgery. Composite outcome measures combine multiple single outcome measures into one. This has several advantages, firstly, composite outcome measures are easier to interpret for clinicians and for patients. For example, textbook outcome (a composite measure describing the event of a radical resection, without intraoperative or postoperative complications, adequate lymph node yield, short hospital stay, no reinterventions, no postoperative mortality and no hospital or ICU readmission) can be interpreted as a completely successful and uneventful operation [86]. Besides, textbook outcome has shown to be related to improved survival rates [88]. Another example is failure to cure (a composite measure describing an R1 or R2 resection, nonresctional/’open–close’ surgery and/or early postoperative mortality), which can be interpreted as an unsuccessful procedure [82,85]. Composite outcome measures also have important statistical advantages in hospital comparison analyses, as the event rate is generally higher than the event rate of single outcome measures. Lastly, composite outcome measures, such as textbook outcome, can be measured over a short period of time. However, textbook outcome is associated with the ultimate oncological outcome of long-term survival, but in contrast to survival can provide short-loop feedback to surgeons which is essential for behavioral change [80]. Recently, international consensus was reached; the textbook outcome parameters were updated and overlapping parameters were removed [89].

### 4.3. Health-Related Quality of Life

Next to measuring the clinical or oncological success (such as textbook outcome), health-related quality of life (HR-QoL) is an important outcome of esophageal cancer surgery. Since overall survival has improved in recent years, HR-QoL has gained increasing interest. HR-QoL can be measured using patient-reported outcome questionnaires. Currently, the most frequently used questionnaires are those from the European Organization for Research and Treatment of Cancer (EORTC) [90]. The EORTC has developed several questionnaires specifically focusing on esophageal or gastroesophageal junction cancer (the EORTC WLW-OES18 and the EORTC WLW-OG25) [91,92,93].

Esophageal cancer surgery severely impacts HR-QoL, but generally, HR-QoL restores to baseline levels after 1 year postoperatively [94]. The occurrence of postoperative complications decreases HR-QoL, which is yet another important plea towards the importance of lowering postoperative complication rates [95,96]. In addition, different types of surgical techniques and approaches influence HR-QoL. For example, minimally invasive transhiatal surgery is associated with inferior HR-QoL compared to minimally invasive transthoracic surgery, and the Ivor Lewis procedure is superior to the McKeown procedure with respect to HR-QoL [97]. This clearly indicates that HR-QoL should be part of patient counseling and preoperative decision making.

## 5. Future Perspectives

### 5.1. Centralization of Esophageal Cancer Surgery

Over recent decades, evidence has been accumulating that higher case-volume results in improved short- and long-term patient outcomes after complex visceral surgery [98,99,100]. Consequently, most western countries have adopted centralization strategies for esophagectomy and other major surgical procedures. Nevertheless, it is important to bear in mind that centralization is a generic term for different concepts which do not necessarily reflect patient needs, but is rather determined by various players, such as physicians, hospitals, Medicare and private payors, policymakers, the media, and national medical societies. As a result, different standards have emerged, and centralization strongly depends on country-specific interpretations and regulations. Thus, regional cutoffs for the annual center volume for esophagectomy range greatly from 7 in Canada to 80 in Denmark, and individual surgeon volume has only been defined in the UK and the US [101].

In this context, it has to be taken into consideration that the simple number of esophagectomies per year may not directly translate into better quality of care, and it has been shown that a positive volume-outcome effect of centralization even turns negative after reaching a certain annual caseload [102]. Therefore, most experts agree that centralization in esophageal surgery not only requires concentration of patients, but also of resources (infrastructure, skilled and experienced personnel, knowledge, and research). In addition, centralization must be accompanied by transparent outcome assessment via national audits and audited multicenter registries. Ideally, outcomes should be measured with well-defined parameters such as complication, mortality and readmission rates, failure to rescue (percentage of patients with a postoperative complication who die as a result of it), textbook outcome (percentage of patients who meet a series of ideal perioperative outcomes) and benchmarking [89,103]. Therefore, as pointed out by D. Low in his landmark publication, specialized upper-GI units with a specific and inherent interest in outcome research are in the best position to meet the future requirements of esophageal cancer surgery centralization [14].

### 5.2. Innovative Intraoperative Techniques

#### 5.2.1. Ischemic Conditioning

Partial gastric devascularization prior to esophagectomy is a relatively new concept in esophageal surgery. The idea of ischemic conditioning (IC) is to optimize the gastric blood flow in preparation for later esophagectomy through preoperative selective occlusion of the left gastric ± short gastric arteries. Devascularization is accomplished by either surgical ligation or interventional embolization [104]. The resulting relative ischemia is expected to resolve via hemodynamic redistribution from the remaining vessels, thus avoiding ischemia during later reconstruction. In addition, partial gastric devascularization may lead to ischemic demarcation, enabling the surgeon to identify the best location for anastomotic reconstruction [105]. Shortcomings of IC include the need for additional resources, increased cost [106], and adhesion formation complicating subsequent gastric mobilization and lymphadenectomy [107]. IC has been investigated in prospective and retrospective cohort studies including several RCT’s [104]. Owing to different technical approaches, it is very difficult to draw conclusions. However, two recent meta-analyses of the current literature did not reveal a significant impact on anastomotic leakage (AL) rates [104]. Therefore, many early advocates have abandoned IC in routine cases and reserve this option for high-risk patients that might benefit from a two-stage approach [108].

#### 5.2.2. Preemptive Endoluminal Vacuum Therapy

Treatment of AL after esophagectomy has considerably progressed in recent years, and surgical revision has been largely replaced by interventional procedures. In addition to stent placement, endoluminal vacuum therapy (EVT) has become the treatment of choice in many specialized centers [109]. In EVT, a polyurethane sponge connected to a hose is brought to the anastomotic area via endoscopy. After vacuum application, the sponge drains the leakage cavity and removes secretions and necrosis, accomplishing an 80–90% healing rate [110].

A novel concept is the application of EVT technology in a preemptive setting (pEVT) with the aim of preventing AL and reducing postoperative morbidity. The clinical efficacy and feasibility of pEVT in patients undergoing minimally invasive esophagectomy have recently shown excellent patient safety outcome parameters [111,112]. In a similar approach, prevention of reflux and protection of the anastomotic area from duodenogastric juices using double-lumen, open-pored, foil vacuum drains have recently been investigated by other groups with promising results [113]. Consequently, this approach is currently being investigated by a randomized controlled trial comparing pEVT with standard postoperative care in high-risk patients undergoing minimally invasive Ivor Lewis esophagectomy [114].

#### 5.2.3. Intraoperative Perfusion Monitoring

Assessment of the viability of the tubulized stomach as an esophageal substitute typically relies on intraoperative subjective evaluation by the surgeon. A healthy gastric tube features rosy-colored tissues, a pulsating vascular arcade, and active bleeding from the staple line. However, depending on vascularization, vasoconstriction, fluid management, catecholamine dosage, and the actual hemodynamic situation, local ischemia is a frequent finding. Therefore, a range of innovative tools for intraoperative assessment of gastric tube viability has been developed over the last years: Laser Doppler flowmetry, near infrared spectroscopy, optical coherence tomography, and laser speckle contrast, infrared thermographic, fluorescence, and hyperspectral imaging. Some of these tools have become commercially available, and preliminary retro- and pro-spective cohort studies [115] and a recent literature review have evidenced some benefit regarding AL rates [116]. Nevertheless, various questions remain unanswered because most technical solutions rely on semiquantitative assessment only, without agreement on perfusion parameters, normal values, and thresholds. Therefore, solid evidence supporting routine intraoperative perfusion monitoring is still pending, and further research is required to explore its clinical potential in this emerging field.

### 5.3. Artificial Intelligence

Artificial Intelligence (AI) in surgery is becoming increasingly popular in surgical literature, and well-recognized surgical units across the world have established their own working groups based on this topic. The Surgical Artificial Intelligence and Innovation Laboratory (SAIIL), consisting of upper gastrointestinal surgeons of Harvard Medical School and Engineers of MIT, has set the first standards to follow [117]. At the same time, medical device companies have increased their investments into this field, resulting in a very broad spectrum of new tools and research areas to be explored in the near future [118]. Computer Vision, Annotation of Surgical Video, and automated phase recognition are examples of first usage of AI in surgery [119,120,121]. The Society of American Gastrointestinal and Endoscopic Surgeons (SAGES) was among the early adopters of AI in surgery and has created an Artificial Intelligence Task Force. Their initial work on annotation standards for surgical video has just been published by an international consensus conference of experts in the field of minimally invasive surgery, medical engineering, and data scientists [122]. Video annotation is a complex procedure and starts with high-quality surgical video that must be ensured in every OR across the world to improve surgical quality.

When looking at AI and surgical therapy of esophageal adenocarcinoma specifically, it becomes clear that the literature is lacking in true surgical reports. Autonomous surgical robots are still far away from reality [123]. Especially in the field of endoscopy, AI-assisted diagnosis of upper-gastrointestinal cancer seems to be a promising first tool that could be of interest for upper-gastrointestinal surgeons, especially in the context of current trials that include active surveillance of esophageal cancer [124]. Endoscopic images of 84,424 patients with upper-gastrointestinal cancer were recently analyzed with an AI-based diagnostic system in a multicenter study in China, resulting in a very high diagnostic accuracy, comparable to expert endoscopists [125]. Future imaging technology may even allow targeted biopsies and other precise and individualized treatment options [126,127].

## 6. Conclusions

With respect to the multimodal treatment of esophageal adenocarcinoma, the main tasks of transthoracic esophagectomy are (1) to achieve a complete resection of the primary tumor with a sufficient clearance of nodal metastasis, (2) to perform this complex surgical procedure without mortality and an acceptable low morbidity, and (3) to offer patients a reasonably high standard of HR-QoL postoperatively. Since the overall caseload remains limited even in specialized centers, an increasing effort is mandatory to develop national and international collaborations for the successful initiation of high-quality surgical trials. All future directives and associated clinical research projects need to address one of these three fields of esophageal cancer surgery to contribute to a general improvement in the patient-related outcome.

## Figures and Tables

**Table 1:** ECCG complication set [76].

Complication Group	Specific Complication
Pulmonary complications	Pneumonia ^1^ Pneumothorax requiring treatment Atelectasis mucous plug requiring a bronchoscopy Pleural effusion in need of additional drainage intervention Respiratory failure leading to reintubation ARDS ^2^ Tracheobronchial injury Acute aspiration Persistent air leak requiring chest tube insertion >10 days after surgery
Gastrointestinal complications	Anastomotic leakage (esophagoenteric leak from anastomosis or staple line or localized conduit necrosis) Necrosis or failure of (gastric) conduit Ileus ^3^ Obstruction of small bowel Complication related to the feeding jejunostomy Complication related to pyloroplasty/pyloromyotomy Clostridium infection Gastrointestinal bleed requiring transfusion or (re)intervention Delayed conduit emptying leading to delayed discharge, intervention, or nasogastric tube insertion >7 days after surgery Pancreatitis Liver dysfunction

^1^: Definition by the Infectious Disease Society of America and the American Thoracic Society; ^2^: Berlin definition; ^3^: Dysfunction of small bowel resulting in delayed enteral feeding.

**Table 2:** ECCG complication definitions [76].

Complication	Definition
Anastomotic leakage	Gastrointestinal defect involving the esophagus, anastomosis, staple line, or conduit in their full thickness. Independent of the method of presentation or identification. Contains three types: Type 1: Local defect treated medically or by dietary restrictions Type 2: Localized defect requiring nonsurgical intervention (i.e., radiological or endoscopical) Type 3: Localized defect requiring surgery
Chyle leakage	Contains three types: Type 1: Treated by dietary restrictions Type 2: Treated by total parenteral feeding Type 3: Requiring a reintervention or reoperation Severity levels are as follows: A = <1 L drain output per day, B = >1 L
Conduit necrosis	Contains three types: Type 1: Focal conduit necrosis identified endoscopically and treated nonsurgically Type 2: Focal conduit necrosis identified endoscopically and not related to free anastomotic or conduit leak, treated with surgery but no esophageal diversion Type 3: Extensive conduit necrosis treated with resection and diversion of the conduit

**Table 3:** Clavien–Dindo complication grading system [77].

Grade	Definition
1	A deviation from the normal postoperative course not requiring pharmacological, radiological, endoscopic and/or surgical intervention. Allowed interventions: antiemetic drugs, antipyretic drugs, analgesics, diuretic drugs, electrolyte suppletion, physiotherapy and bedside wound opening for wound infections.
2	A deviation requiring pharmacological treatment with other drugs than mentioned under grade 1, blood transfusion and/or total parenteral nutrition.
3	A deviation requiring endoscopic, radiologic or surgical intervention 3A: reintervention not under general anesthesia 3B: reintervention under general anesthesia
4	Life treathening complication requiring intensive care unit admission 4A: single organ dysfunction (including dialysis) 4B: multiorgan
5	Mortality of the patient
